# Localization and Interaction between Kinin B1 Receptor and NADPH Oxidase in the Vascular System of Diabetic Rats

**DOI:** 10.3389/fphys.2017.00861

**Published:** 2017-10-31

**Authors:** Youssef Haddad, Réjean Couture

**Affiliations:** Department of Pharmacology and Physiology, Faculty of Medicine, Université de Montréal, Montréal, QC, Canada

**Keywords:** bradykinin B1 receptor, endothelium, macrophages, NOX1, NOX2, diabetes mellitus, VSMC

## Abstract

Kinin B1 receptor (B1R) enhanced superoxide anion (${\text{O}}_{2}^{•-}$) production in the vasculature of diabetic rats. This study investigates the induction and distribution of B1R in diabetic blood vessels and addresses the hypothesis that B1R is co-localized with NADPH oxidase (NOX1 and NOX2) and produces its activation via protein kinase C (PKC). Diabetes was induced in rats with streptozotocin (STZ 65 mg.kg^−1^, i.p.). Two weeks later, the production of ${\text{O}}_{2}^{•-}$ was measured in aorta rings in response to the B1R agonist (Sar[D-Phe^8^]-des-Arg^9^-BK, 20 μM) by the method of lucigenin-enhanced chemiluminescence. Various inhibitors were added (10 μM) to block PKC_total_ (Ro-31-8220), PKCβ1/2 (LY333531), or NADPH oxidase (Diphenyleneiodonium). The cellular localization of B1R was studied in the aorta, popliteal artery, and renal glomerulus/arteries by immunofluorescence and confocal microscopy with markers of endothelial cells (anti-RECA-1), macrophages (anti-CD11), vascular smooth muscle cells (anti-SMA), and NADPH oxidase (anti-NOX1 and NOX2). Although B1R was largely distributed in resistant vessels, it was sparsely expressed in the aorta's endothelium. The greater basal production of ${\text{O}}_{2}^{•-}$ in STZ-diabetic aorta was significantly enhanced by the B1R agonist (15–45 min). The peak response to the agonist (30 min) was inhibited by all inhibitors. Immunofluorescent staining for B1R, NOX1, and NOX2 was significantly increased in endothelial cells, vascular smooth muscle cells, and macrophages of STZ-diabetic aorta on which they were found co-localized. Data showed that B1R enhanced ${\text{O}}_{2}^{•-}$ by activating vascular NADPH oxidase through PKCβ1/2. This was substantiated by the cellular co-localization of B1R with NOX1 and NOX2 and opens the possibility that B1R-enhanced oxidative stress is derived from vascular and infiltrating immune cells in diabetes.

## Introduction

Sustained hyperglycemia-induced oxidative stress exposes individuals to chronic, low-grade inflammation, and contributes to diabetes and its complications. Oxidative stress occurs when the production of reactive oxygen species (ROS) overwhelms the anti-oxidative defense (Johansen et al., 2005; Gao and Mann, 2009). Hyperglycemia and the accumulation of metabolites in the glycolysis pathway, notably advanced glycation end-products, activate NADPH oxidase through protein kinase C (PKC) and thereby contribute to the major source of ROS (such as superoxide anion, ${\text{O}}_{2}^{•-}$) in vascular cells (He and King, 2004; Gao and Mann, 2009; Chen et al., 2014). ROS are known to activate a variety of signaling pathways and transcription factors, notably the nuclear factor NF-kB, which mediates the transcription of several pro-inflammatory genes, especially pro-inflammatory cytokines such as TNF, IL-1, IL-6, IL-8 (Cai et al., 2003; Cave et al., 2006), chemokines [e.g., MCP-1 (monocyte chemoattractant protein 1), also known as CCL2] and cellular adhesion molecules such as intercellular adhesion molecule-1 (ICAM-1), vascular cell adhesion molecule-1 (VCAM-1), integrins, selectins that increase macrophages and leukocytes infiltration in the vasculature leading to endothelial dysfunction and vascular disease (He and King, 2004; Feng et al., 2005; Goldberg, 2009; Gleissner, 2015).

Kinins are vasoactive peptides that play an important role in cardiovascular homeostasis, pain, and inflammation. These autacoids activate two G-protein-coupled receptors (GPCR) named B1R and B2R. While B2R is constitutive and mediates most effects of bradykinin (BK) including inflammation, vasodilation, and capillary leakage, B1R is weakly expressed in physiological situation, yet it is induced by pro-inflammatory cytokines (IL-1β, TNF) and by ROS during diabetes through the NF-kB pathway (Couture and Girolami, 2004; Couture et al., 2014). In a rat model of insulin resistance induced by high glucose feeding, B1R increased the production of ${\text{O}}_{2}^{•-}$ via the activation of NADPH oxidase while B1R antagonism prevented the enhanced basal production of ${\text{O}}_{2}^{•-}$ by NADPH oxidase and the upregulation of inducible nitric oxide synthase (iNOS) in the aorta (Dias et al., 2010; Dias and Couture, 2012b). Given that B1R can activate iNOS through Gαi, Gβγ and Src-dependent activation of the ERK/MAP kinase pathway to generate high level of NO (Kuhr et al., 2010), one can suggest that peroxynitrite (ONOO^−^) generated from the combination of NO and ${\text{O}}_{2}^{•-}$ (Johansen et al., 2005) can contribute to the detrimental effect of B1R and to the reduction of endothelium-derived NO bioavailability in diabetes (Couture et al., 2014; Haddad and Couture, 2016). The production of ROS by B1R-induced activation of NADPH oxidase could represent a putative mechanism by which B1R antagonism reversed the auto-induction of B1R and its pro-inflammatory effects in models of diabetes (Dias et al., 2010; Dias and Couture, 2012a,b; Pouliot et al., 2012). A recent concept involving B1R in the propagation of inflammation has also been proposed in human vascular disease, which consists in the transfer to target organ recipient cells of a massive release of circulating leukocyte-derived microvesicles bearing functional B1R (Kahn et al., 2017).

The multiple forms of NADPH oxidases are emerging as important targets for prevention of vascular oxidative stress and cardiovascular diseases (Sun et al., 2016). The NOX isoforms of NADPH oxidases are transmembrane proteins that transfer electrons through biological membranes. NADPH oxidase catalyzes the transfer of electrons from NADPH to oxygen via its catalytic subunits to generate ROS (${\text{O}}_{2}^{•-}$ or H_2_O_2_). Four NOX isoforms are expressed in the vascular wall, including NOX1 (endothelial cells and VSMC), NOX2 (endothelial cells, adventitial fibroblasts, and leukocytes such as monocytes, macrophages, and platelets), NOX4 (endothelial cells, VSMC, and adventitial fibroblasts), and NOX5 (endothelial and VSMC–not expressed in rodents) (Wendt et al., 2005; Drummond et al., 2011). Because of their marked enhanced activities, NOX1 and NOX2 represent the most important superoxide-generating enzymes in diabetes and vascular disease. The subsequent formation of peroxynitrite (ONOO^−^) resulting from the binding of ${\text{O}}_{2}^{•-}$ and NO reduces NO bioavailability and activates pro-inflammatory signaling pathways in addition to causing irreversible damage to macromolecules including proteins, lipids, and DNA, thereby disrupting crucial cell signaling pathways and promoting cell death. On the other hand, NOX4 produces H_2_O_2_ and may provide protective function to the vascular wall by increasing NO bioavailability and suppressing cell death pathways; H_2_O_2_ does not react with NO and may even act as an endothelium-derived relaxing factor (Drummond and Sobey, 2014).

The objective of present study was two-fold; first, to determine whether PKC is involved in the activation of NADPH oxidase by B1R and if the two main isoforms of NADPH oxidase (NOX1 and NOX2) are upregulated and co-localized with B1R on vascular smooth muscle cells (VSMC), endothelium, and infiltrating macrophages in the diabetic thoracic aorta; second, to compare the distribution of B1R in the aorta (conductance vessel) with that of resistance arteries (popliteal and renal afferents/efferents and glomeruli) in diabetic rats.

## Materials and methods

### Animal care and ethics approval

All animal care and experimental procedures complied with the Use of Laboratory Animals and were approved by the Université de Montréal's Committee on Ethics in the Care and Use of Laboratory Animals (protocol 15–013) http://www.cdea.umontreal.ca in accordance with the guiding principles as enunciated by the Canadian Council on Animal Care. Animal studies are reported in compliance with the ARRIVE guidelines (Kilkenny et al., 2010; McGrath and Lilley, 2015).

### Experimental procedures

Male Sprague-Dawley rats (200–225 g; Charles River Laboratories, Saint-Constant, QC, Canada) were housed two per cage, under standard conditions of temperature (22.5°C) and humidity (42.5%), on a 12 h/12 h light-dark cycle and allowed free access to normal chow diet (Charles River Rodent) and tap water. Four days after their arrival, rats were made diabetic by a single injection of streptozotocin (STZ) freshly prepared (65 mg.kg^−1^; i.p.; Cayman Chemical, Michigan, USA). Age-matched controls were injected with sterile saline (0.9%). Blood glucose concentration was determined with a glucometer (Accu-Chek Aviva; Roche Diagnostics, Laval, QC, Canada) in blood samples obtained from the tail vein, in non-fasting animals. Only STZ-treated rats whose blood glucose concentration was higher than 20 mM were considered as diabetic. At the end of 2 weeks, the thoracic aorta, popliteal artery, and renal cortex were removed under isoflurane anesthesia and kept frozen at −80°C.

### Superoxide anion measurement

Superoxide anion production was measured from aortic slices using the lucigenin-enhanced chemiluminescence method as described previously (Ohara et al., 1993; Munzel et al., 1995). Briefly, small rings of 3–5 mm from STZ and control aorta were pre-incubated at 37°C in Krebs-Hepes buffer (saturated with 95% O_2_ and 5% CO_2_) for 30 min and then transferred to a glass scintillation vial containing 5 μM of lucigenin in 2 ml for the determination of basal ${\text{O}}_{2}^{•-}$ levels. The chemiluminescence was recorded every minute for 10 min in a dark room at room temperature in a liquid scintillation counter (Wallac 1409, Turku, Finland). The B1R agonist (A-B1R) Sar[D-Phe^8^] des-Arg^9^-BK (Drapeau et al., 1993) was added to tissue samples after the 30 min pre-incubation period and lucigenin counts were determined at time 0, 15, 30, and 45 min. The activation of NADPH oxidase in the samples was assessed by adding 0.1 mM NADPH to the vials 30 min before adding the B1R agonist. In a separate series of experiments, inhibitors (10 μM) of NADPH oxidase (DPI, Diphenyleneiodonium), PKC_total_ (Ro-31-8220) and PKCβ1/2 (LY333531) were added 30 min prior to the addition of the B1R agonist and lucigenin counts were determined at time 0 min (baseline) and at the peak time response (30 min) to the B1R agonist. The background was counted using a vial with no tissue but with the solution. The final value expressed as cpm.mg^−1^ of fresh tissue was calculated as follows: (tissue value − background value) ÷ weight of fresh tissue.

### Cellular localization of B1R, NOX1, and NOX2 by immunofluorescence

After sacrifice of rats, blood vessels and renal cortex were immediately frozen in 2-methylbutane cooled at −45 to −55°C with liquid nitrogen and kept at −80°C. They were mounted in an OCT (optimal cutting temperature) compound embedding medium for frozen tissue specimens (Fisher healthcare #4585) and serially cut into 20 μm-thick coronal sections (or 10 μm for renal cortex) on a cryostat and stored at −80°C. Sections were mounted on Fisherbrand Superfrost Plus Microscope Slides (Fisher Scientific #12-550-15) and put in vacuum jar overnight to ensure their adhesion to the slides. Next day, sections were fixed in paraformaldehyde (PFA 4%) for 2 min, washed for 5 min × three times with PBS (phosphate buffered saline 1X). Slides were incubated for 1 h with a blocking buffer PBS supplemented with 10% bovine serum albumin (BSA Roche diagnostic #10735086001) and 10% goat serum (Cedarlane CL1200-100) + Triton X-100 0.3% (Sigma Aldrich # T9284) to prevent non-specific labeling. Primary antibodies were diluted (1:100) in blocking buffer, put over the slides overnight, washed for 5 min × three times with PBS on the next day, reincubated for 2 h with a secondary antibody diluted (1:200) in blocking buffer, washed with PBS 5 min × three times then the sections were covered by fluorescence mounting media (Glycerol/n-propyl gallate mounting medium prepared in lab) and the coverslip was fixed by applying a little nail varnish to its edges. Images were examined with Olympus IX-81 inverted fluorescence microscope; digitized RGB images were transferred to a computer and analyzed using NIH ImageJ 1.36b Software (NIH, Bethesda, MD, USA). Semi-quantification of immunofluorescence staining intensity (white vs. gray) was made on 10 randomly selected surface areas of a minimum of four blood vessel sections per rat from four controls and four STZ-diabetic rats. Background intensity (gray intensity) was subtracted from each individual value.

Images (single focal plane or Z-stack) were also captured with an oil immersion objective 40X x Plan-Apochromat objective (1.4 NA) on a Zeiss LSM 800 laser-scanning confocal microscope (Carl Zeiss, Jena, Germany). For confocal optical sectioning, sequences of images were taken along the optical axis with adequate increments (1.00 μm).

### Drugs and antibodies

Sar[D-Phe^8^] des-Arg^9^-BK was synthetized by Bio Basic Inc. Biotechnology Company (Markham, ON, Canada). Streptozotocin (STZ), NADPH (Nicotinamide Adenine Dinucleotide Phosphate Hydrogen), DPI (Diphenyleneiodonium), inhibitors of PKC_total_ (Ro-31-8220, No: 13334), and PKCβ1/2 (LY333531, No: 13964) were all purchased from Cayman Chemical (Michigan, USA). The following primary antibodies were used: home-made rabbit Anti-B1R (Lin et al., 2010; Lacoste et al., 2013); mouse Anti-RECA-1 (NB-10064647, Novus Biologicals, Littleton, CO, USA); mouse Anti-rat CD11b/c recognition of macrophages (Cedarlane CL042AP, Burlington, ON, Canada); mouse Anti-alpha smooth muscle Actin (abcam ab7817, Toronto, ON, Canada) while goat Anti-NOX1 (SC-5821) and goat Anti-NOX2 (Anti gp91-phox, SC-5827) were from Santa Cruz Biotechnology, CA, USA. Secondary antibodies were obtained from Life Technology (Thermo Fisher Scientific, MA USA): Donkey Anti-mouse, Alexa Fluor 555 (A-31570), Donkey Anti-rabbit, Alexa Fluor 488 (A-21206), Donkey Anti-goat, Alexa Fluor 633 (A-21082).

### Statistical analysis

Data are expressed as the mean ± SEM, and *n* represents the number of rats. The statistical analysis was performed using GraphPad prism software (GraphPad Software, Version 5, La Jolla, CA, USA). Data and statistical analysis comply with the recommendations on experimental design and analysis in pharmacology (Curtis et al., 2015). Statistical significance was determined with Student's *t*-test for unpaired samples or with the one-way ANOVA followed by the Bonferroni test for multiple comparisons when F achieved *P* < 0.05 and there was no significant variance in homogeneity. A *P*-value of ≤ 0.05 was considered statistically significant.

## Results

### Effect of B1R agonist on superoxide anion production

**Figure 1:**
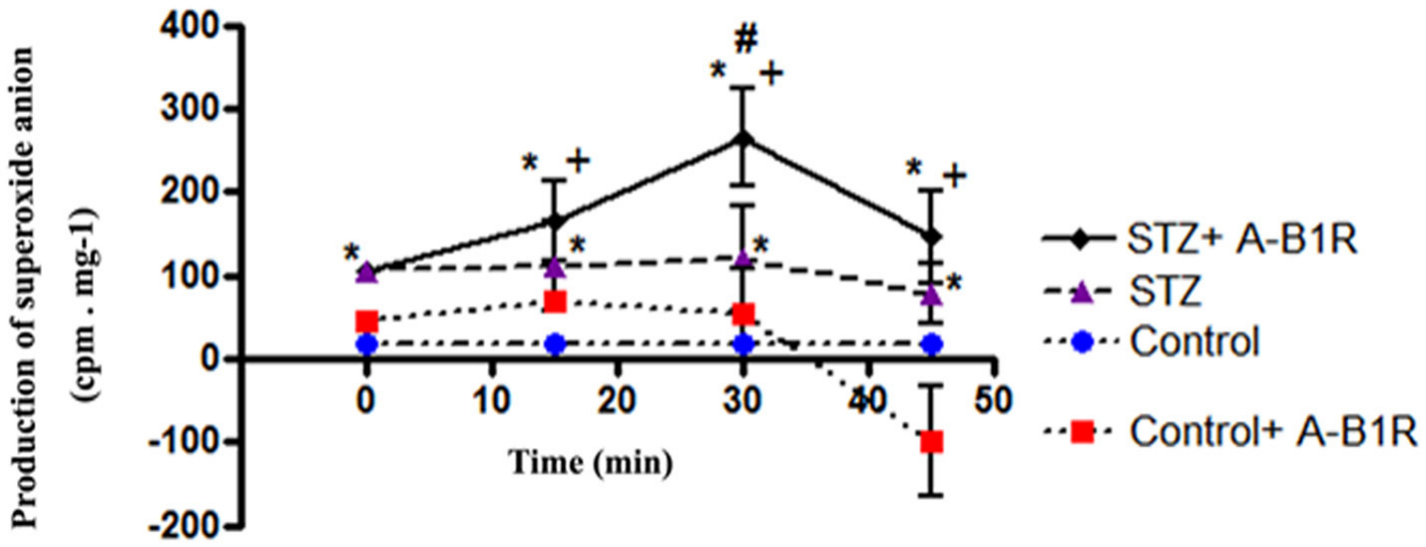
Production of superoxide anion in control and STZ-aortic slices in the absence and presence of the B1R agonist (A-B1R) Sar[D-Phe^8^] des-Arg^9^-BK (20 μM) at 0, 15, 30, and 45 min using the lucigenin-enhanced chemiluminescence method. Data are mean ± SEM obtained from five rats per group. *P < 0.01 STZ vs. Control; *P < 0.01 STZ + A-B1R vs. Control; ^+^*P* < 0.05 STZ + A-B1R vs. Control + A-B1R at 15 min or ^+^*P* < 0.01 at 30 and 45 min; ^#^*P* < 0.01 STZ + A-B1R vs. STZ.

**Figure 2:**
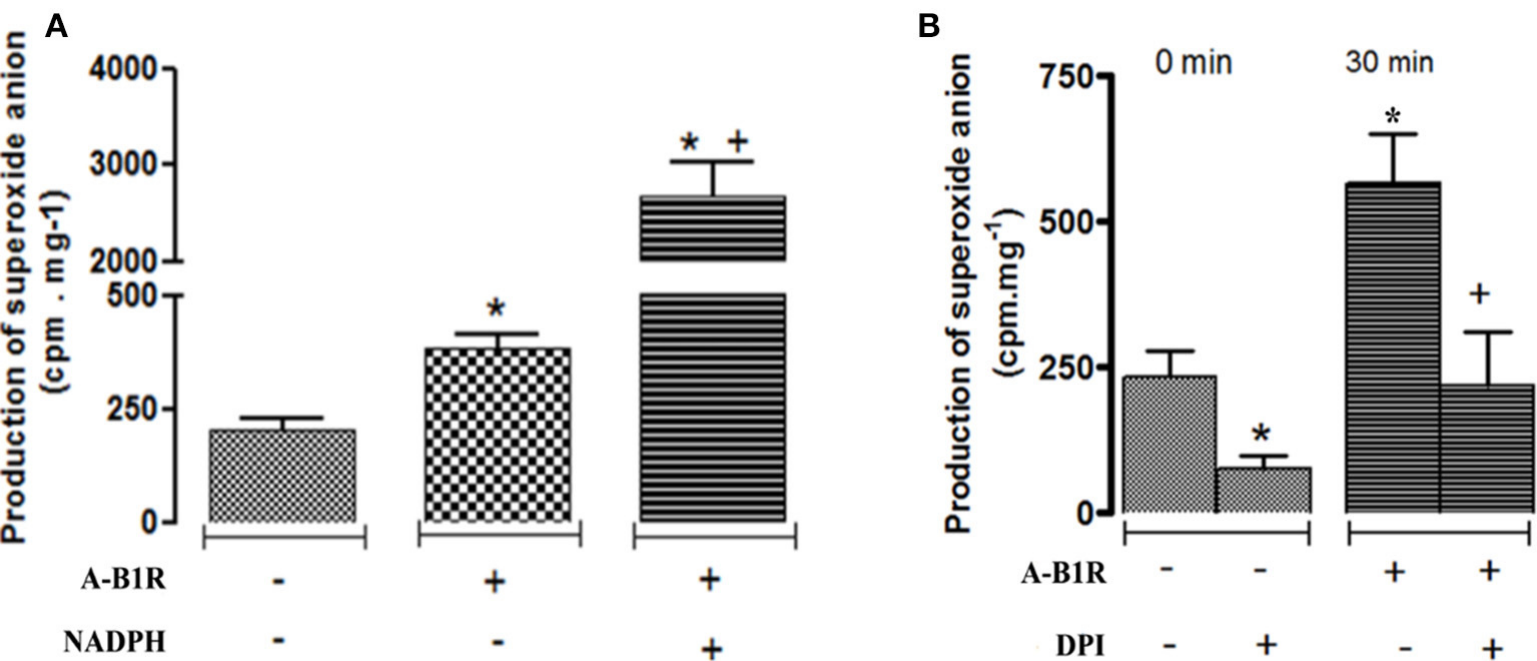
Production of superoxide anion evoked by the B1R agonist (A-B1R) Sar[D-Phe^8^] des-Arg^9^-BK (20 μM) at 0 min (–) and 30 min (+) in STZ-aortic slices in the absence (–) and presence (+) of **(A)** NADPH (0.1 mM), the substrate of NADPH oxidase, or **(B)** DPI (diphenyleneiodonium, 10 μM), an inhibitor of NADPH oxidase. Data are mean ± SEM obtained from five rats per group. In **(A)** *P < 0.05 A-B1R vs. Baseline; *P < 0.001 A-B1R + NADPH vs. Baseline; ^+^*P* < 0.001 A-B1R + NADPH vs. A-B1R. In **(B)** *P < 0.05 DPI vs. Baseline; *P < 0.01 A-B1R vs. Baseline; ^+^*P* < 0.01 A-B1R + DPI vs. A-B1R.

Basal production of ${\text{O}}_{2}^{•-}$ was significantly enhanced in STZ-aorta in comparison to control-aorta during the 45-min incubation period (Figure 1). The addition of the B1R agonist (Sar[D-Phe^8^] des-Arg^9^-BK, 20 μM) at time 0 significantly amplified the production of ${\text{O}}_{2}^{•-}$ in STZ-aorta at 15, 30 (peak effect) and 45 min post-agonist stimulation. In contrast, B1R agonist failed to significantly affect ${\text{O}}_{2}^{•-}$ production during the same period in control-aorta. To assess the activity of NADPH oxidase in the production of ${\text{O}}_{2}^{•-}$ evoked by the B1R agonist, its enzyme substrate NADPH was added to the vials. As depicted in Figure 2A, the maximal production of ${\text{O}}_{2}^{•-}$ measured by the B1R agonist at 30 min was significantly boosted in the presence of NADPH (0.1 mM). To confirm the involvement of NADPH oxidase, diphenyleneiodonium (DPI, 10 μM), an inhibitor of NADPH oxidase (Massart et al., 2013; Song et al., 2015) was added to the vials 30 min prior to the addition of the B1R agonist. Data show that both the basal production of ${\text{O}}_{2}^{•-}$ at time 0 min and that induced by 30 min stimulation with the B1R agonist were significantly blocked by DPI, confirming that ${\text{O}}_{2}^{•-}$ derived from NADPH oxidase (Figure 2B).

**Figure 3:**
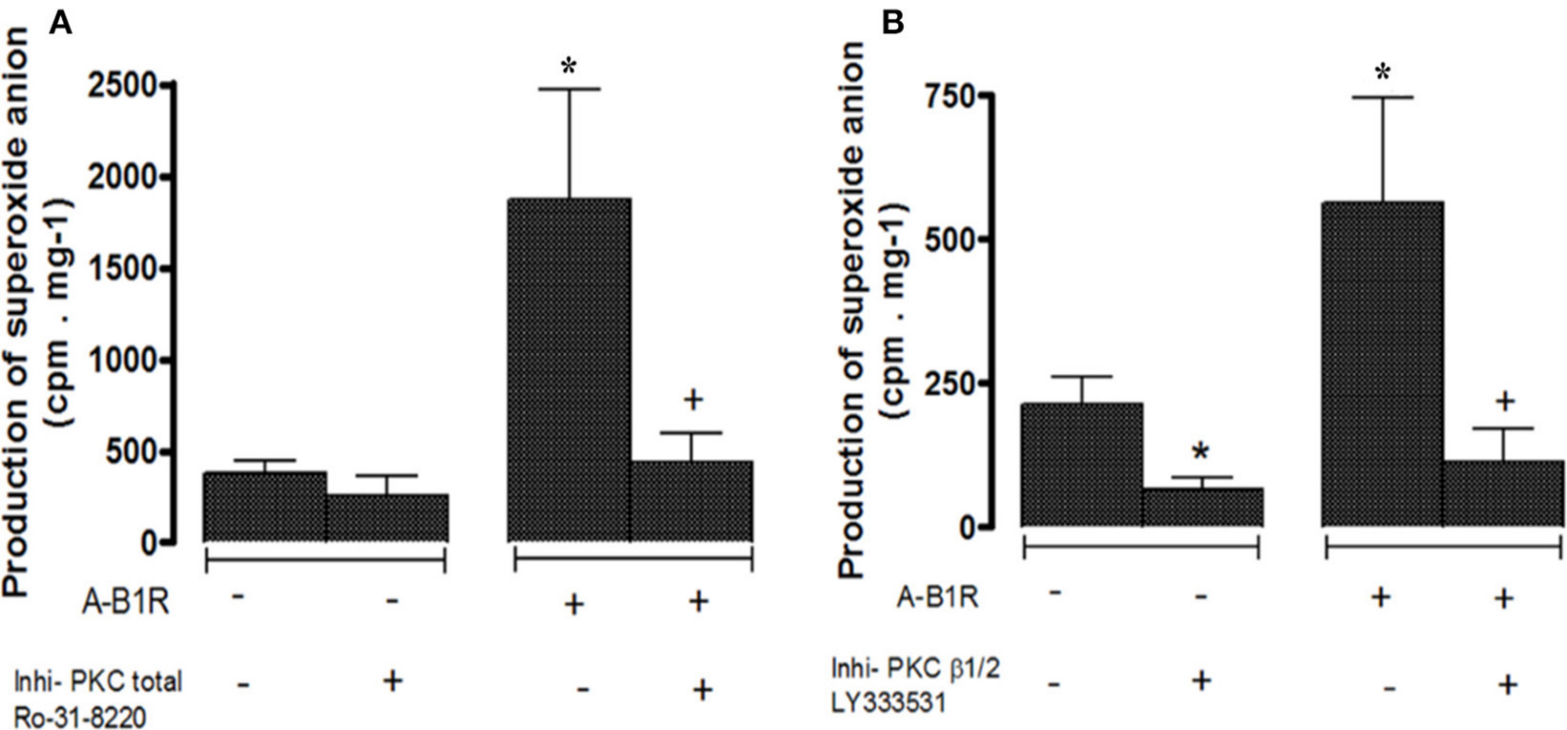
Production of superoxide anion evoked by the B1R agonist (A-B1R) Sar[D-Phe^8^] des-Arg^9^-BK (20 μM) at 0 min (–) and 30 min (+) in STZ-aortic slices in the absence (–) and presence (+) of **(A)** PKC_total_ inhibitor (Ro-31-8220, 10 μM), or **(B)** PKCβ1/2 inhibitor (LY333531, 10 μM). Data are mean ± SEM obtained from five rats per group. In **(A)** *P < 0.001 A-B1R vs. Baseline; ^+^*P* < 0.001 A-B1R + Ro-31-8220 vs. A-B1R. In **(B)** *P < 0.05 LY333531 vs. Baseline;*P < 0.01 A-B1R vs. Baseline; ^+^*P* < 0.01 A-B1R + LY333531 vs. A-B1R.

To determine the involvement of PKC in B1R-induced NADPH oxidase activation, experiments were carried out in the presence of an inhibitor of PKC_total_ (Ro-31-8220) or a selective inhibitor of PKCβ1/2 (LY333531) (Gray et al., 2013) added at 10 μM 30 prior to stimulation with the B1R agonist in STZ-aorta. As shown in Figure 3, the maximal production of ${\text{O}}_{2}^{•-}$ induced by Sar[D-Phe^8^] des-Arg^9^-BK (20 μM) at 30 min was significantly blocked by either inhibitor confirming the contribution of PKCβ1/2 in the activation of NADPH oxidase by the B1R agonist. Data also suggest the contribution of PKCβ1/2 in the basal production of ${\text{O}}_{2}^{•-}$ induced by NADPH oxidase in STZ-aorta as evidenced by the significant reduction caused by the inhibitor in the absence of B1R agonist.

### Cellular localization of B1R in thoracic aorta

**Figure 4:**
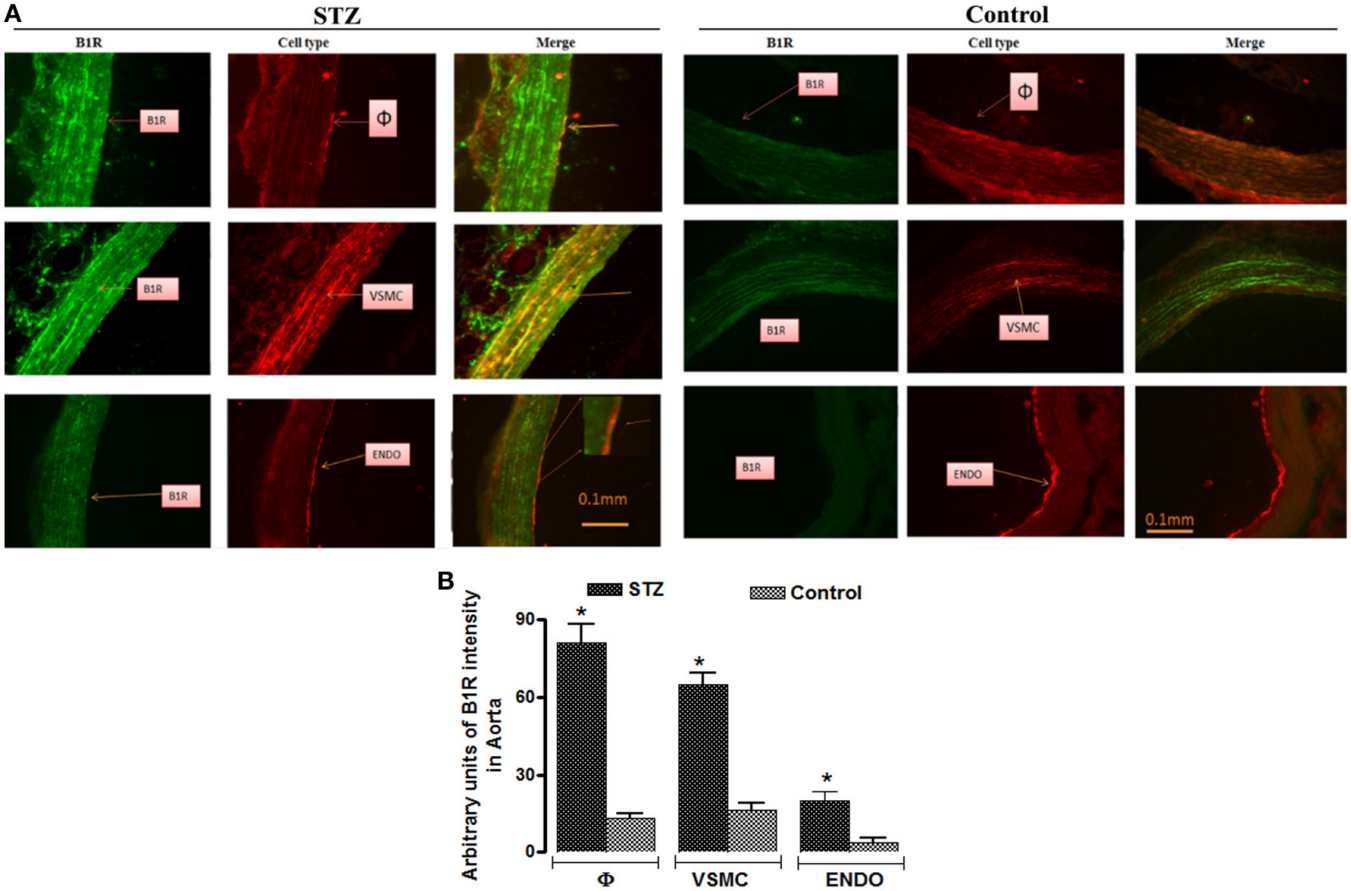
Representative fluorescent photomicrographs **(A)** of STZ and control aortic sections showing immunofluorescent staining for B1R (Green color), macrophages (φ, Red color), vascular smooth muscle cells (VSMC, Red color), and endothelial cells (Endo, Red color). Merge pictures (as Orange-Yellow color) show the co-localization of B1R with macrophages and VSMC, and some endothelial cells in STZ-aorta. No co-localization was seen in control-aorta between B1R and the three cell types. Scale bars = 0.1 mm. Data are representative of a minimum of four aortic sections per rat from four controls and four STZ-diabetic rats. Bar Graph **(B)** illustrates semi-quantitative B1R immunofluorescent intensity in macrophages, VSMC and endothelial cells in STZ- and Control-aortae. Data are mean ± SEM obtained from four rats per group; *P < 0.0001 STZ vs. Control.

**Figure 5:**
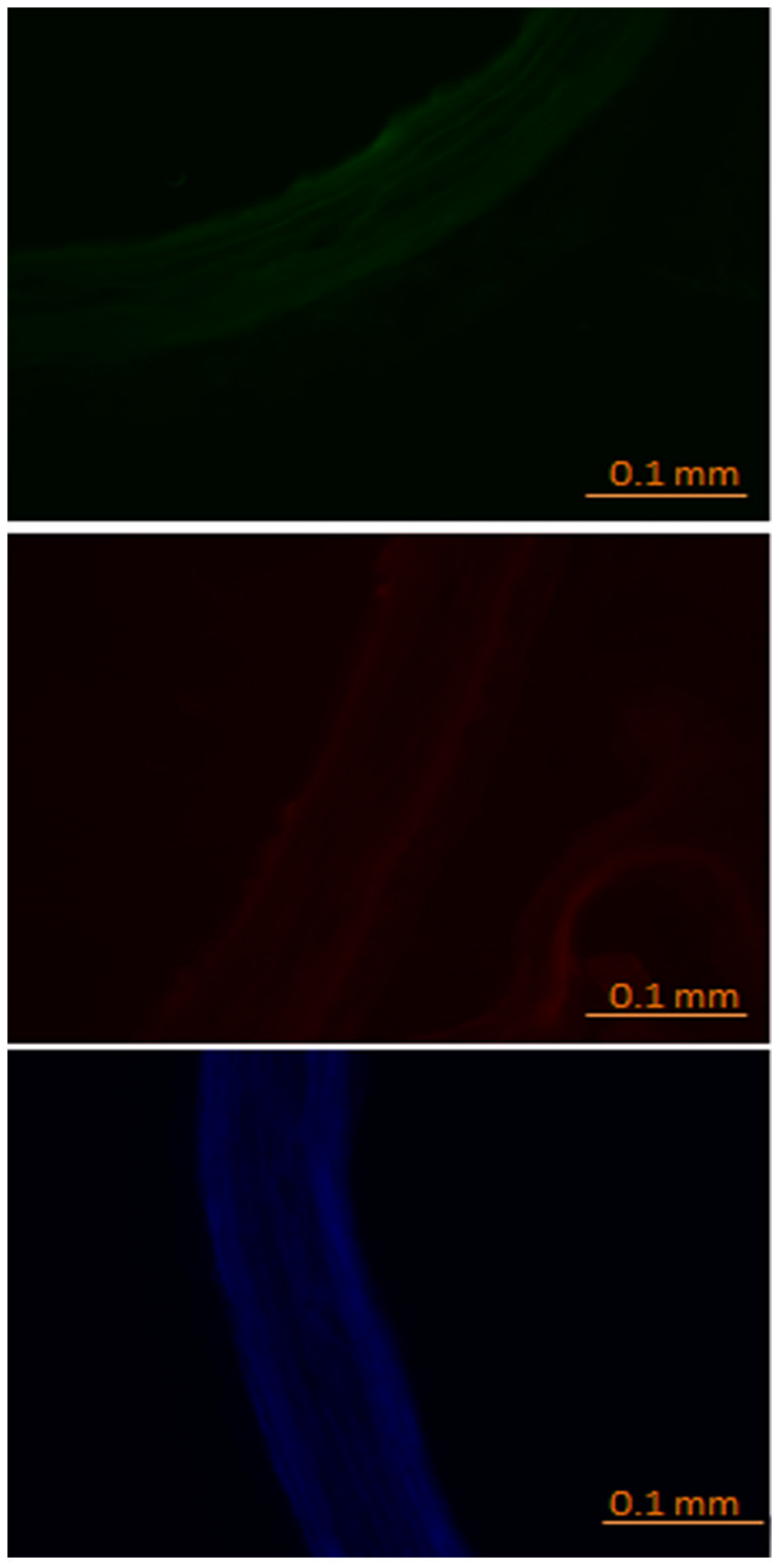
Autofluorescence of STZ-aorta was shown for Alexa Fluor 488-Anti Rabbit (Green color, **A**), Alexa Fluor 555-Anti Mouse (Red color, **B**), Alexa Fluor 633-Anti Goat (Blue color, **C**). The primary antibodies were omitted. Scale bars = 0.1 mm. Data are representative of a minimum of four aortic sections from two STZ-diabetic rats.

Representative pictures of STZ- and control- aortae labeled for B1R (tagged with anti-B1R in green color), macrophages (tagged with anti-CD11 in red color), VSMC (tagged with anti-SMA in red color), and endothelial cells (tagged with anti-RECA-1 in red color) are presented in Figure 4A. B1R labeling was relatively weak in control-aorta and merge labeling (as orange-yellow color) was not seen in control-aorta revealing no localization of B1R on the three cell types. In contrast, B1R labeling was quite striking on macrophages and VSMC of STZ-aorta. The co-localization of B1R on the endothelium of STZ-aorta was less pronounced and seen only on a few cells. Semi-quantitative values of B1R immunofluorescent intensity in each cell type are also shown in Figure 4B. B1R labeling was relatively weak in control-aorta on the three cell types, yet it was significantly greater in STZ-aorta (macrophages › VSMC › endothelium). As control, **t**he omission of primary antibodies produced a small autofluorescence background in STZ-aortic sections in the presence of secondary antibodies (Figure 5). Thus, this confirms that B1R is hardly detectable in control-aorta after subtracting the green autofluorescence.

### Cellular localization of NOX1 and NOX2 in thoracic aorta

**Figure 6:**
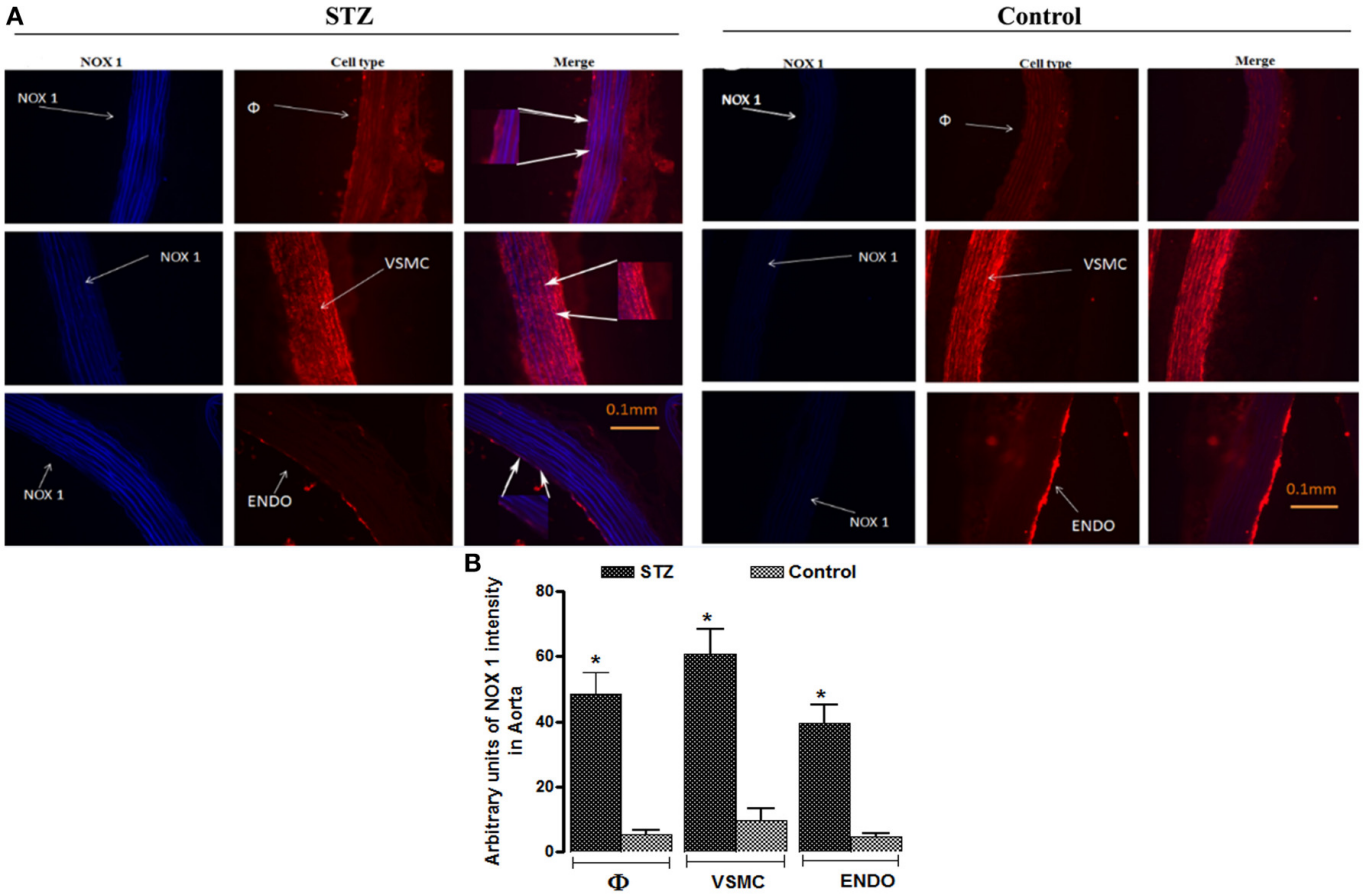
Representative fluorescent photomicrographs **(A)** of STZ and control aortic sections showing immunofluorescent staining for NOX1 (Blue color), macrophages (φ, Red color), vascular smooth muscle cells (VSMC, Red color), and endothelial cells (Endo, Red color). While merge labeling (as Blue-Purple color) was absent in control-aorta, it was obvious in the STZ-aorta on each cell type. Scale bars = 0.1 mm. Data are representative of a minimum of four aortic sections per rat from four controls and four STZ-diabetic rats. Bar Graph **(B)** illustrates semi-quantitative NOX1 immunofluorescent intensity in macrophages, VSMC, and endothelial cells in STZ- and Control-aortae. Data are mean ± SEM obtained from four rats per group; *P < 0.0001 STZ vs. Control.

**Figure 7:**
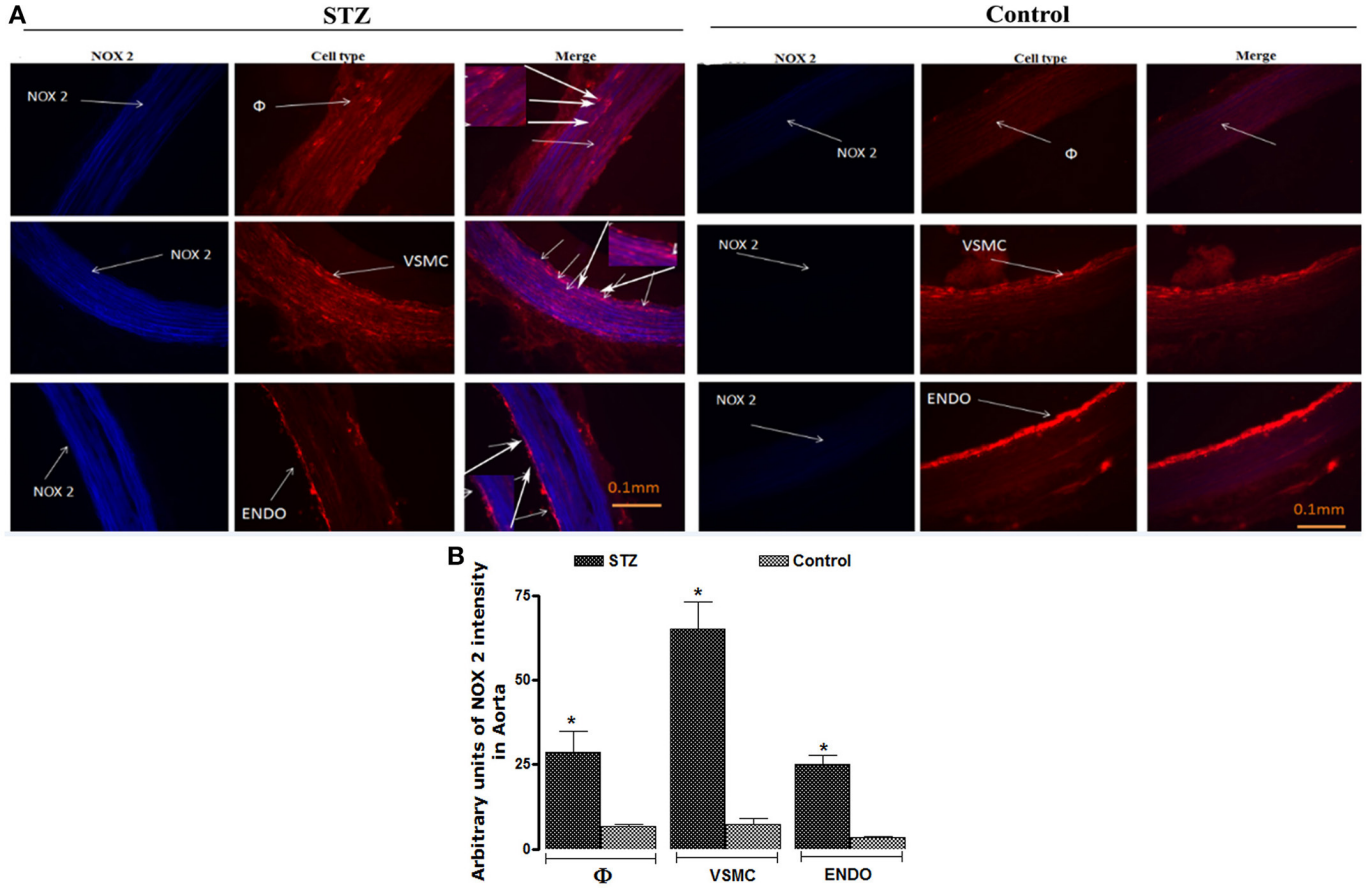
Representative fluorescent photomicrographs **(A)** of STZ and control aortic sections showing immunofluorescent staining for NOX2 (Blue color), macrophages (φ, Red color), vascular smooth muscle cells (VSMC, Red color), and endothelial cells (Endo, Red color). While merge labeling (as Blue-Purple color) was small in control-aorta, it was obvious in the STZ-aorta on each cell type. Scale bars = 0.1 mm. Data are representative of a minimum of four aortic sections per rat from four controls and four STZ-diabetic rats. Bar Graph **(B)** illustrates semi-quantitative NOX2 immunofluorescent intensity in macrophages, VSMC, and endothelial cells in STZ- and Control-aortae. Data are mean ± SEM obtained from four rats per group; *P < 0.0001 STZ vs. Control.

Representative immunofluorescent pictures of STZ- and control- aortae are depicted for NOX1 (Figure 6A) and NOX2 (Figure 7A) (tagged with anti-NOX1 and anti-NOX2 in blue color), macrophages (tagged with anti-CD11 in red color), VSMC (tagged with anti-SMA in red color), and endothelial cells (tagged with anti-RECA-1 in red color). Data revealed that NOX1 and NOX2 were barely detectable in control-aorta (background is autofluorescence as seen in Figure 5) while their immuno-expression was quite pronounced in STZ-aorta. Merge labeling (as blue-purple color) shows evidence of co-localization of NOX1 and NOX2 with macrophages, VSMC, and endothelial cells in STZ-aorta. In contrast, macrophages, VSMC, and endothelial cells show no evidence of labeling for NOX1 (Figure 6A) and NOX2 (Figure 7A) in control-aorta. Semi-quantitative values of NOX1 and NOX2 immunofluorescent intensity in each cell type are also shown in Figures 6B, 7B, respectively. Semi-quantitatively, NOX1 and NOX2 immunostaining intensities were significantly higher in all cell types of STZ-aorta, particularly in VSMC.

### Co-localization of B1R with NOX1 and NOX2 in thoracic aorta

**Figure 8:**
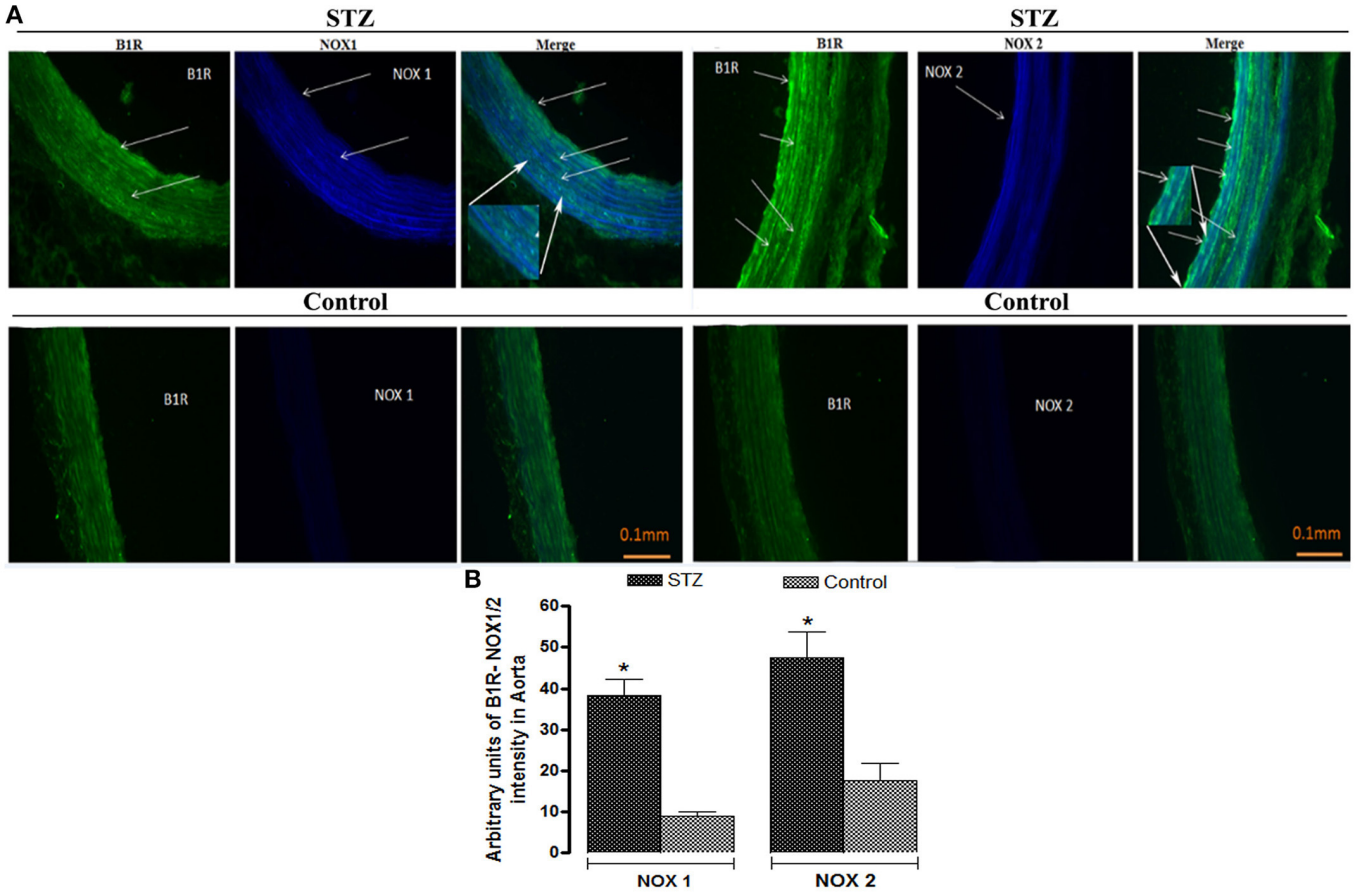
Representative fluorescent photomicrographs **(A)** of STZ and control aortic sections showing immunofluorescent staining for B1R (Green color), NOX1 or NOX2 (Blue color). While merge labeling (as Turquoise color dots) was not seen in control-aorta, it occurred in STZ-aorta that highlighted co-localization of B1R with NOX1/2. Scale bars = 0.1 mm. Data are representative of a minimum of four aortic sections per rat from four controls and four STZ-diabetic rats. Bar Graph **(B)** illustrates semi-quantitative immunofluorescent intensity for B1R with NOX1 and NOX2 in STZ- and Control-aortae. Data are mean ± SEM obtained from four rats per group; *P < 0.0001 STZ vs. Control.

**Figure 9:**
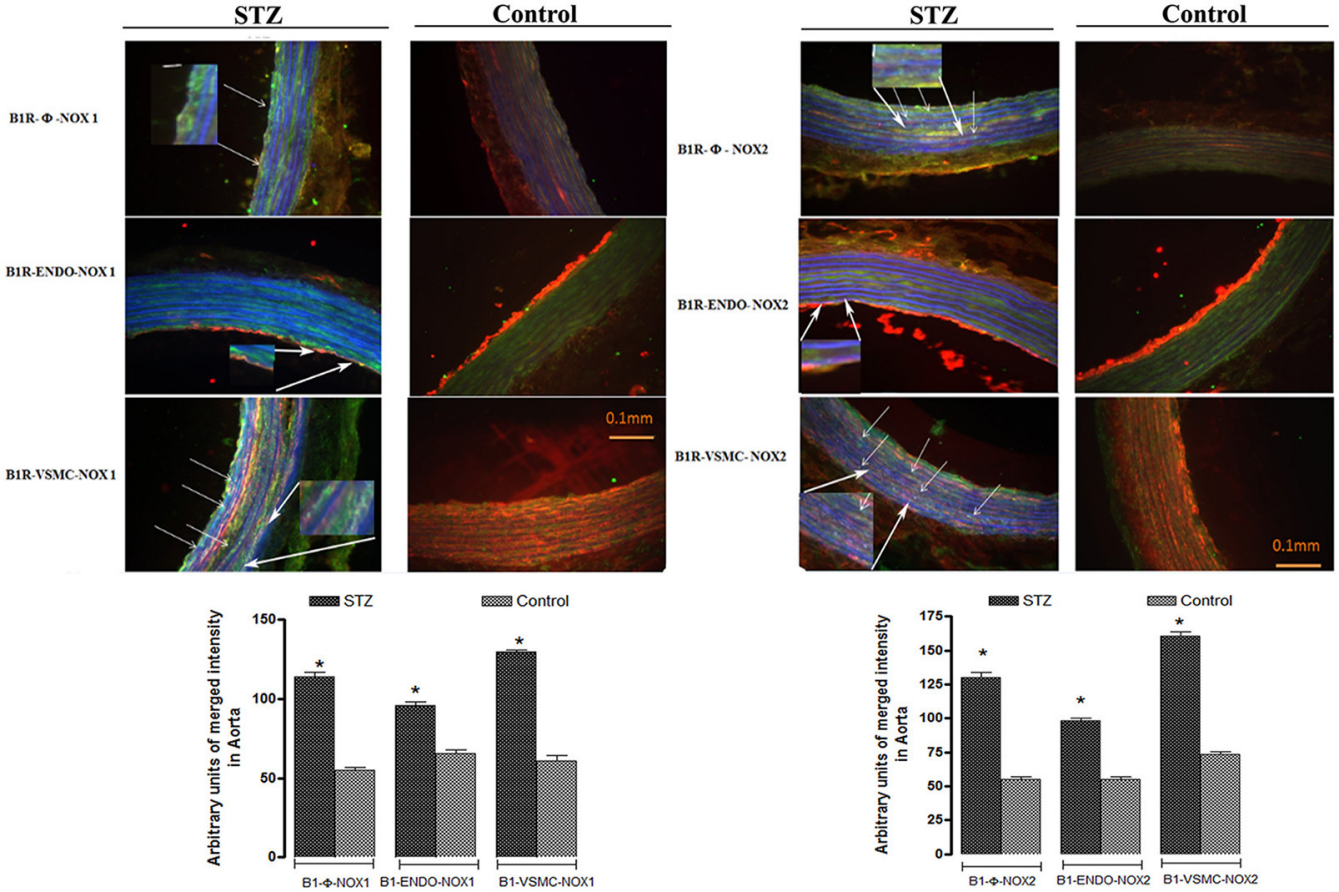
Representative fluorescent photomicrographs of STZ and control aortic sections showing merge immunofluorescent staining (as White color dots) between B1R and NOX1 (**Upper-Left**) or B1R and NOX2 (**Upper-Right**) on the same cell type (macrophages and VSMC and to some extent on endothelial cells) in STZ-aorta. Such co-localization did not occur in control-aorta. Scale bars = 0.1 mm. Data are representative of a minimum of four aortic sections per rat from four controls and four STZ-diabetic rats. Bottom Bar Graphs illustrate semi-quantitative immunofluorescent intensity for B1R with NOX1 **(Left)** and NOX2 **(Right)** on the same cell type in STZ- and Control-aortae. Data are mean ± SEM obtained from four rats per group; *P < 0.0001 STZ vs. Control.

**Figure 10:**
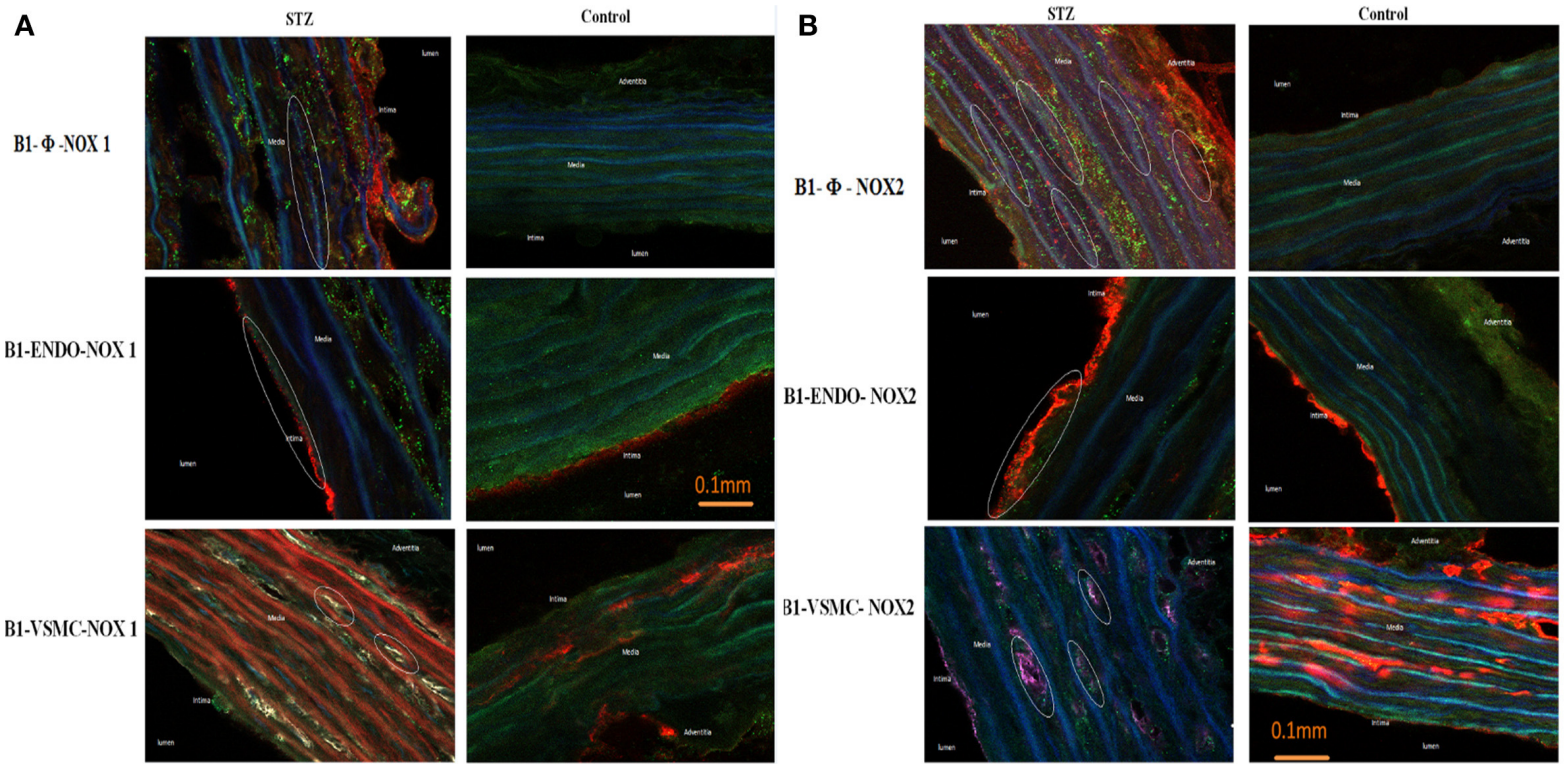
Microphotographs of immunolocalization of B1R with NOX1 **(A)** and NOX2 **(B)** on macrophages, endothelial cells and VSMC by confocal microscopy (40 X). Shown are immunolabeling for B1R (green), macrophages (φ, Red color), endothelial cells (Endo, Red color), vascular smooth muscle cells (VSMC, Red color), and NOX1 or NOX2 (Blue color) in STZ- and Control-aorta. Note that B1R co-localized (as White color dots) with NOX1 and NOX2 on macrophages, VSMC, and rarely on endothelial cells in STZ-aorta. Triple immunocolocalization was not seen in Control-aorta. Data are representative of a minimum of four aortic sections per rat from four Controls and four STZ-diabetic rats.

As B1R, NOX1, and NOX2 were found on VSMC, infiltrating macrophages and to some extent to endothelial cells in STZ-aorta, it remains to determine whether B1R co-localizes with NOXs on the same elements. Figure 8A illustrates representative immunofluorescent pictures of STZ- and control- aortae for NOX1 and NOX2. Whereas, merge immunostaining (as turquoise color dots) of B1R (green color) and NOX1/NOX2 (blue color) was not seen in control-aorta, it was highly detectable in STZ-aorta. The level of coexpression of B1R with NOX1 and B1R with NOX2 was highly significant in STZ-aorta when compared to control-aorta (Figure 8B). Furthermore, in triple labeling, co-localization of B1R with NOX1 (Figures 9, 10A) and B1R with NOX2 (Figures 9, 10B) was highly significant on the same cell type. While merge labeling (white color dots) was absent in control-aorta, it was markedly present on VSMC and infiltrating macrophages and more rarely on endothelial cells of STZ-aorta.

### Cellular localization of B1R in popliteal artery

**Figure 11:**
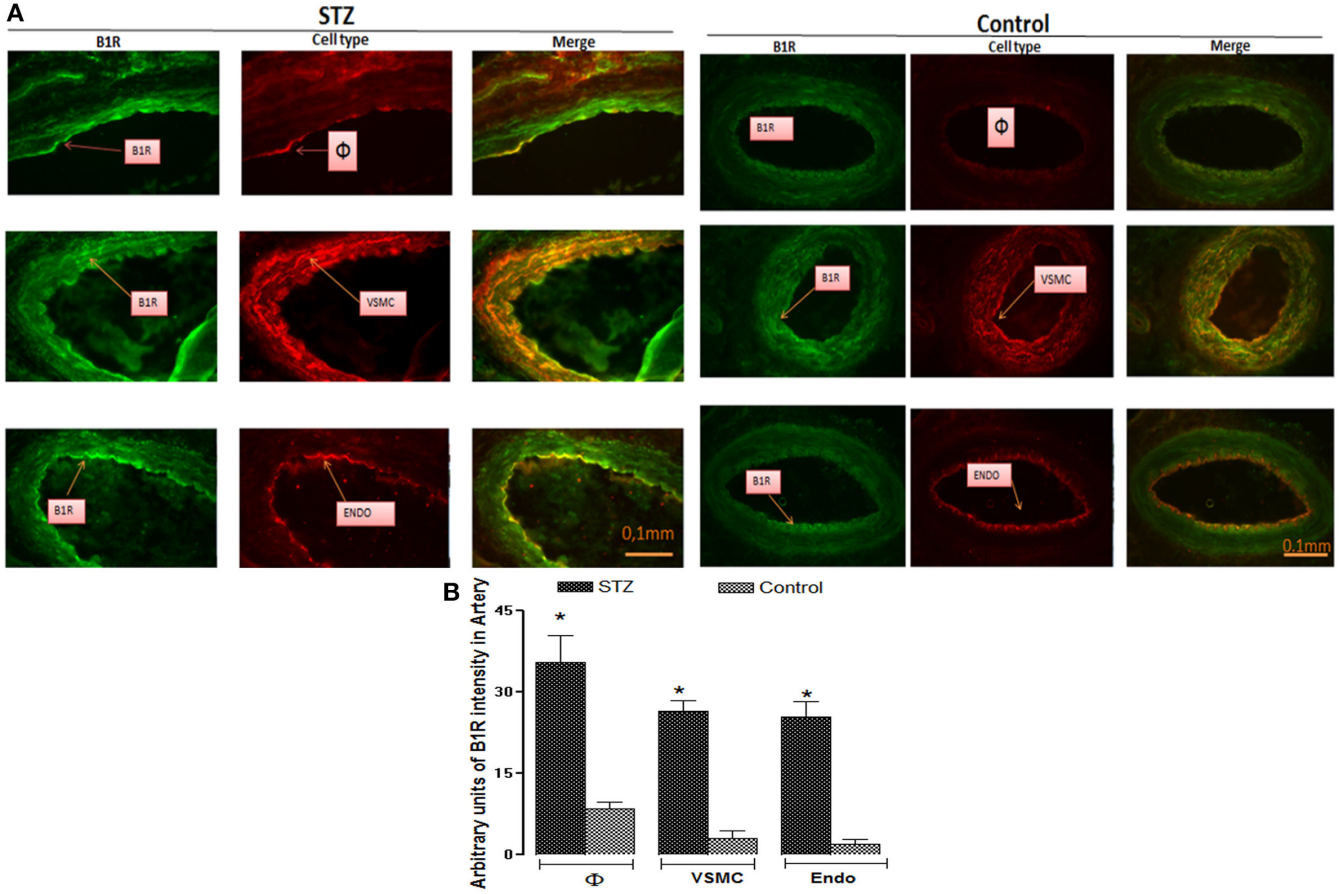
Representative fluorescent photomicrographs **(A)** of STZ and control popliteal sections showing immunofluorescent staining for B1R (Green color), macrophages (φ, Red color), vascular smooth muscle cells (VSMC, Red color), and endothelial cells (Endo, Red color). Merge pictures (as Orange-Yellow color) show the co-localization of B1R on macrophages, VSMC, and endothelial cells in STZ-popliteal artery. B1R was also slightly expressed on VSMC and endothelial cells in the control resistance artery. Scale bars = 0.1 mm. Data are representative of a minimum of four popliteal sections per rat from four controls and four STZ-diabetic rats. Bar Graph **(B)** illustrates semi-quantitative B1R immunofluorescent intensity in macrophages, VSMC, and endothelial cells in STZ- and Control-popliteal artery. Data are mean ± SEM obtained from four rats per group; *P < 0.0001 STZ vs. Control.

The distribution of B1R was assessed in the popliteal artery as a prototype of resistance artery (Figure 11A). Red labeling was depicted to identify macrophages, VSMC, and endothelial cells, yet infiltrating macrophages were absent in the control artery. B1R intensity of labeling (green color) was significantly greater in STZ-artery than in control-artery on each cell type (Figures 11A,B). Importantly, B1R was found co-localized (yellow color) with infiltrating macrophages, VSMC and endothelial cells in the STZ-artery while weak co-localization was also found in VSMC and endothelial cells in control-artery. Contrarily to the STZ-aorta, localization of B1R on the endothelium was more pronounced in STZ-popliteal artery (Figures 11A,B).

### Cellular localization of B1R in renal arteries and glomeruli

**Figure 12:**
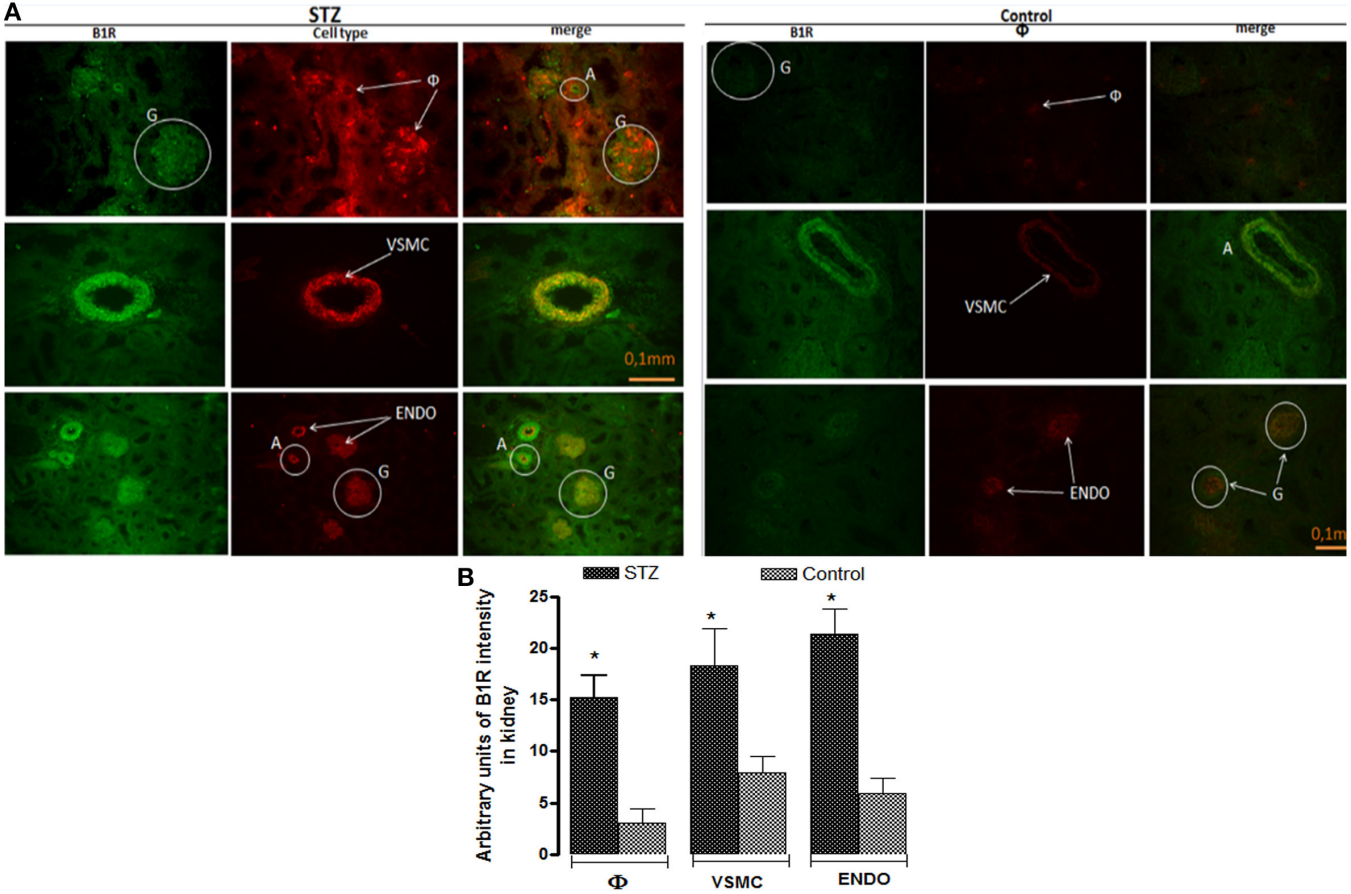
Representative fluorescent photomicrographs **(A)** of STZ and control renal cortex showing immunofluorescent staining for B1R (Green color), macrophages (φ, Red color), vascular smooth muscle cells (VSMC, Red color), and endothelial cells (Endo, Red color). Merge pictures (as Orange-Yellow color) show the co-localization of B1R on macrophages, VSMC, and endothelial cells in STZ-arteries (A) and/or glomeruli (G). B1R was not coexpressed in the same control structures except in glomeruli. Scale bars = 0.1 mm. Data are representative of a minimum of four renal sections per rat from four controls and four STZ-diabetic rats. Bar Graph **(B)** illustrates semi-quantitative B1R immunofluorescent intensity in macrophages, VSMC and endothelial cells in STZ- and Control-renal cortex. Data are mean ± SEM obtained from four rats per group; *P < 0.0001 STZ vs. Control.

The localization of B1R was studied in small afferent/efferent arteries surrounding glomeruli in the renal cortex of STZ and control rats for comparison with larger arteries such as the thoracic aorta and popliteal artery (Figure 12A). The labeling of B1R (green) and macrophages (red) was almost absent in control renal cortex while it was quite striking in STZ renal cortex showing co-localization of B1R with infiltrating macrophages in glomeruli and surrounding tissue. Likewise, B1R was co-localized with VSMC in larger renal arteries and endothelial cells in small afferent/efferent arteries and glomeruli of STZ. No apparent co-localization was found in those control renal structures, except in control glomeruli. This was ascertained semi-quantitatively in which B1R intensity of labeling was significantly higher in endothelium, VSMC and infiltrating macrophages of STZ renal cortex compared with control renal cortex (Figure 12B).

## Discussion

This study showed that activation of B1R, a Gαq-protein coupled receptor, stimulates PKCβ1/2 to enhance the production of ${\text{O}}_{2}^{•-}$ through NADPH oxidase in the thoracic aorta of STZ-diabetic rats. Inhibitors of NADPH oxidase (DPI), PKC (Ro-31-8220), and PKC β1/2 (LY333531) prevented the increased production of ${\text{O}}_{2}^{•-}$ induced by the B1R agonist (Sar[D-Phe^8^]-des-Arg^9^-BK) in isolated STZ-aorta. Also, the addition of NADPH boosted the activation of NADPH oxidase induced by the B1R agonist. In contrast, the B1R agonist was ineffective in control-aorta, which is consistent with the low basal expression levels of B1R and NOX1/2 detected by immunofluorescence in non-diabetic control aorta. Importantly, this study provided the first evidence that B1R and NADPH oxidase are co-localized on VSMC, infiltrating macrophages and endothelial cells in STZ-diabetic blood vessels, thereby contributing to enhanced vascular oxidative stress. The upregulation of B1R in STZ blood vessels is likely a consequence of the higher basal level of oxidative stress (${\text{O}}_{2}^{•-}$) as documented earlier in STZ-retina (Abdouh et al., 2008). The cellular distribution of B1R on VSMC, endothelial cells (mainly on resistance vessels vs. aorta), and macrophages is consistent with its primary role in vasomotor tonus control (contractility/relaxation), vascular permeability, and cellular inflammation.

The present study demonstrated a substantial increase in the expression of NOX1 and NOX2 associated with increased NADPH oxidase activity in STZ-diabetic aorta. The two-fold upregulation of NOX1 with no changes in NOX4 was also reported in 8 weeks STZ-diabetic rat aorta (Wendt et al., 2005). Earlier studies have reported enhanced protein and gene expression of B1R in vessels and peripheral organs of STZ-diabetic rats (Pouliot et al., 2011, 2012; Tidjane et al., 2015, 2016) and in models of type 2 diabetes and insulin resistance (Ismael et al., 2008; Dias et al., 2010; Dias and Couture, 2012a; Midaoui et al., 2015; Haddad and Couture, 2016). B1R activation increased the basal production of ${\text{O}}_{2}^{•-}$ while B1R antagonism decreased it in the vasculature of diabetic rats along with a reduction of vascular inflammation and B1R expression (Dias et al., 2010; Dias and Couture, 2012b). The present study suggests that B1R augments oxidative stress in diabetes by enhancing NADPH oxidase activity in a PKCβ1/2-dependent mechanism on VSMC, endothelial cells and infiltrating macrophages. The pro-oxidative effect of B1R is believed to activate the transcription nuclear factor NF-κB known to further amplify and perpetuate the expression of B1R and other pro-inflammatory mediators (cytokines, iNOS, adhesion molecules, and so on) (Couture et al., 2014). The present data also highlight the likelihood contribution of infiltrating macrophages in addition to VSMC and endothelial cells in this pro-oxidative and pro-inflammatory vicious cycle in diabetic vascular tissues. This is congruent with the findings that prolonged treatment with B1R antagonists prevented the infiltration of macrophages and other immune cells in retina, pancreas, and adipose tissue, a mechanism which can contribute to the therapeutic effects of B1R antagonism in diabetic retinopathy, insulin resistance, and other diabetic complications (Dias et al., 2010; Dias and Couture, 2012b; Pouliot et al., 2012; Tidjane et al., 2015, 2016; Talbot et al., 2016). The expression of B1R on macrophages in STZ-aorta, popliteal artery, and in the vicinity of renal glomeruli is reminiscent to B1R expression documented on macrophages and immune cells infiltrating the STZ-diabetic pancreas (Tidjane et al., 2016). This is keeping with previous findings showing the expression of B1R on macrophages and T lymphocytes (Bockmann and Paegelow, 2000; Couture et al., 2001) and with the up-regulation of B1R in endothelial cells, foamy macrophages, inflammatory cells, fibroblasts, and VSMC in atheromatous patients (Raidoo et al., 1997) and human brain endothelial cells exposed to interferon-gamma (Prat et al., 2000).

The protein Rac-GTP is required for NOX1 and NOX2 activity (George et al., 2013). PKC-mediated activation of Rac is an established mechanism of NADPH oxidase activation in a variety of cell types (Bedard and Krause, 2007). Upon activation, there is an exchange of GDP for GTP on Rac leading to phosphorylation of the cytosolic p47^phox^ subunit provoking conformational changes allowing interaction with p22^phox^. The movement of p47^phox^ brings with it the other cytoplasmic subunits, p67^phox^ and p40^phox^, to form the active NOX1/2 enzyme complex. The active enzyme complex transports electrons from cytoplasmic NADPH to extracellular oxygen to generate ${\text{O}}_{2}^{•-}$ (Bedard and Krause, 2007). Increases in PKCβ and PKCβ1 activities have been shown to account for much of the elevated total PKC activity in hyperglycemic human brain microvascular endothelial cells and both act upstream to NADPH oxidase to increase the apoptotic effect of oxidative stress (${\text{O}}_{2}^{•-}$) that impact negatively on blood-brain barrier (Shao and Bayraktutan, 2014). This is congruent with our findings that inhibition of total PKCβ and PKCβ1/2 abolished ${\text{O}}_{2}^{•-}$ production mediated by NADPH oxidase activity following B1R activation in STZ-diabetic thoracic aorta.

## Conclusion

Pharmacological evidence is provided that activation of B1R increases the oxidative stress through a mechanism involving the stimulation of NADPH oxidase and PKCβ1/2 in the STZ-diabetic aorta. Moreover, the hypothesis is confirmed that B1R is upregulated and co-localized with NADPH oxidase (NOX1 and NOX2) in VSMC and infiltrating macrophages in STZ-diabetic aorta. Whereas, B1R was highly expressed on endothelial cells in small resistance arteries, its occurrence was weak in large conductance artery of STZ-diabetic rats. These findings further support a primary role for kinin B1R in vascular inflammation and oxidative stress that may be of importance in the treatment of vascular diseases and hyperglycemia-induced cell apoptosis, particularly in the setting of diabetes.

## Author contributions

YH and RC conceived and designed the experiments. YH performed the experiments, analyzed the data, and drafted the paper. RC supervised the study, edited, and wrote the final version of the manuscript. All authors approved the final manuscript and contributed to editorial changes in the manuscript.

### Conflict of interest statement

The authors declare that the research was conducted in the absence of any commercial or financial relationships that could be construed as a potential conflict of interest.

## Abbreviations

BK: bradykinin
B1R: bradykinin type 1 receptor
DPI: diphenyleneiodonium
H_2_O_2_: hydrogen peroxide
iNOS: inducible nitric oxide synthase
IL-1, IL-6, IL-8: interleurkin-1,-6, and -8
ICAM-1: intercellular adhesion molecule-1, φ, macrophages
MCP-1: monocyte chemoattractant protein 1
NADPH: nicotnamide adenine dinucleotide phosphate hydrogen
NO: nitric oxide
NF-kB: nuclear factor kappa-light-chain-enhancer of activated B cells
PKC: protein kinase C
ONOO^−^: peroxynitrite
ROS: reactive oxygen species
${\text{O}}_{2}^{•-}$: superoxide anion
STZ: streptozotocin
TNF: tumor necrosis factor-alpha
VSM: vascular smooth muscle cells
VCAM-1: vascular cell adhesion molecule-1.
